# Brand switching and toxic chemicals in cigarette smoke: A national study

**DOI:** 10.1371/journal.pone.0189928

**Published:** 2018-01-11

**Authors:** Jennifer R. Mendel, Sabeeh A. Baig, Marissa G. Hall, Michelle Jeong, M. Justin Byron, Jennifer C. Morgan, Seth M. Noar, Kurt M. Ribisl, Noel T. Brewer

**Affiliations:** 1 Lineberger Comprehensive Cancer Center, University of North Carolina, Chapel Hill, NC, United States of America; 2 Department of Health Behavior, Gillings School of Global Public Health, University of North Carolina, Chapel Hill, NC, United States of America; 3 School of Media and Journalism, University of North Carolina, Chapel Hill, NC, United States of America; Universidade do Estado do Rio de Janeiro, BRAZIL

## Abstract

**Introduction:**

US law requires disclosure of quantities of toxic chemicals (constituents) in cigarette smoke by brand and sub-brand. This information may drive smokers to switch to cigarettes with lower chemical quantities, under the misperception that doing so can reduce health risk. We sought to understand past brand-switching behavior and whether learning about specific chemicals in cigarette smoke increases susceptibility to brand switching.

**Methods:**

Participants were US adult smokers surveyed by phone (*n* = 1,151, probability sample) and online (*n* = 1,561, convenience sample). Surveys assessed whether smokers had ever switched cigarette brands or styles to reduce health risk and about likelihood of switching if the smoker learned their brand had more of a specific chemical than other cigarettes. Chemicals presented were nicotine, carbon monoxide, lead, formaldehyde, arsenic, and ammonia.

**Results:**

Past brand switching to reduce health risk was common among smokers (43% in phone survey, 28% in online survey). Smokers who were female, over 25, and current “light” cigarette users were more likely to have switched brands to reduce health risks (all *p* < .05). Overall, 61–92% of smokers were susceptible to brand switching based on information about particular chemicals. In both samples, lead, formaldehyde, arsenic, and ammonia led to more susceptibility to switch than nicotine (all *p* < .05).

**Conclusions:**

Many US smokers have switched brands or styles to reduce health risks. The majority said they might or would definitely switch brands if they learned their cigarettes had more of a toxic chemical than other brands. Brand switching is a probable unintended consequence of communications that show differences in smoke chemicals between brands.

## Introduction

More than 7,000 chemical compounds (constituents) have been identified in cigarettes and cigarette smoke,[1] at least 69 of which are known carcinogens.[2] In addition to cancer, cigarette smoke chemicals also cause cardiovascular, respiratory, reproductive, and developmental problems.[3] Smokers have heard of relatively few of these chemicals but have an interest in learning more about them.[4, 5] The 2009 Family Smoking Prevention and Tobacco Control Act requires the US Food and Drug Administration (FDA) to produce a list of harmful and potentially harmful chemicals in cigarette smoke. This list must also include the quantities of each chemical by brand and sub-brand. The Act requires that this information be publicly displayed “in a format that is understandable and not misleading to a lay person.”[6]

Researchers have raised concerns that disclosing quantities of chemicals by brand and sub-brand could mislead smokers into thinking some cigarettes are safer than others,[7, 8] even though there is not scientific evidence that any type of combusted cigarettes are substantially safer than any others.[9] Some smokers mistakenly believe that switching cigarette brands or styles, such as switching to cigarettes advertised as “light,” can reduce their health risk from smoking.[10] By 1998, so-called “light” cigarette brands comprised 82% of the market.[11] Brand switching is a problematic behavior because it is sometimes associated with compensatory smoking behavior (i.e., smoking more cigarettes per day or inhaling more deeply)[12] and a lower likelihood of quitting smoking.[10] In light of the Tobacco Control Act’s requirement to make information about quantities of chemicals in cigarette brands available to the public, more research is needed to understand the potential effects of disclosing this information, particularly on brand switching. We therefore sought to investigate: 1) demographic correlates of past brand-switching behavior, and 2) smokers’ interest in switching brands if they learned that their cigarettes have a lot more of a particular chemical than other cigarettes.

## Methods

### Participants

We recruited a national probability sample of 5,014 US adults (ages 18 and older) from September 2014 to June 2015 to participate in a phone survey using a combination of random digit dial landline and cell phone frames. Detailed information on sampling and methodology are available elsewhere.[13] Additionally, in December 2014, we recruited an online convenience sample of 4,137 adults via Amazon Mechanical Turk (www.mturk.com) to participate in an online survey.[14] The analytic sample for the current study includes only current cigarette smokers (*n* = 1,151 in the phone survey, *n* = 1,561 in the online survey). We obtained informed consent for participation at the time of enrollment. The Institutional Review Board at the University of North Carolina approved the study procedures.

### Procedures and measures

The phone and online surveys included descriptive measures as well as an experiment (S1 Appendix). The surveys used the same measures aside from minor modifications to the online items as needed based on mode. We cognitively tested new survey items for clarity with 14 adult smokers and nonsmokers.[15]

The survey assessed past brand switching with the question, “Have you ever switched to another cigarette brand or style to reduce your health risk?” (no, coded as 0; yes, coded as 1). The survey[13] also assessed participant demographics, including age, sex, sexual orientation, race, ethnicity, education, income, and poor mental health (i.e., describing mental health as “fair” or “poor”).[16] The survey also measured numeracy, current smoking, e-cigarette use, and quit intentions. Numeracy, the ability to understand numeric information, was measured with the following item, “In general, which of these numbers shows the biggest risk of getting a disease?” Response options were “one in 100,” “one in 1,000,” or “one in 10.”[17] We coded the correct answer as indicating high numeracy (1) and others as indicating lower numeracy (0). We defined current smokers as those who had smoked at least 100 cigarettes in their lifetime and currently smoked every day or some days.[18] We further classified them into “every day” and “some days” smokers. We defined participants who said they smoke “light,” “mild,” “ultra-light,” “gold,” or “silver” cigarettes as smokers of “light” cigarettes, and those who smoke “regular,” “red,” or “full-flavor” cigarettes as smokers of regular cigarettes. Smokers also indicated whether they had ever used an e-cigarette or other vaping devices and whether they planned to quit smoking within the next 6 months (i.e., quit intentions).

In the between-subjects experiment, we randomized participants to respond to items about 1 of 6 chemicals: nicotine, carbon monoxide, lead, formaldehyde, arsenic, or ammonia. The outcome was susceptibility to brand or style switching (both within and between brands), assessed with the question, “What if you learned that the cigarettes you smoke have a lot more [chemical] than other cigarettes? How likely would you be to switch to another cigarette brand or style?” The response options were “you might switch,” “you’d definitely switch,” or “you wouldn’t switch.” We dichotomized this variable, combining “might” and “would definitely” switch (susceptible, coded as 1) compared to “wouldn’t switch” (not susceptible, coded as 0). The 6 chemicals chosen were among the most well-known to the public[14, 19] of FDA’s abbreviated list of harmful and potentially harmful chemicals.[20] Randomization successfully created groups that did not differ with respect to participant characteristics, including past brand switching, as evidenced by only 2 out of 80 statistically significant associations between experimental condition (i.e., chemical) and selected demographic characteristics across phone and online surveys.

### Data analysis

We analyzed data from the phone and online surveys separately. We first used unadjusted logistic regression to identify demographic and tobacco product use characteristics that were correlates of past brand switching. We then used adjusted logistic regression to examine whether each correlate remained statistically significant after accounting for all other statistically significant correlates of past switching in either sample. Next, we examined the effect of chemical on susceptibility to brand switching with unadjusted logistic regression. We chose nicotine as a reference group because it elicits less discouragement from smoking than other chemicals[19, 21] and therefore is likely to have a smaller effect on brand switching. All analyses were unweighted except for those examining correlates of past brand switching in the phone survey, which we weighted to provide nationally representative estimates for the corresponding odds ratios.[13] Data analyses used SAS v 9.4, a critical alpha of .05 and two-tailed tests.

## Results

The average age of participants was 43 (*SD* = 15) in the phone survey and 35 (*SD* = 13) in the online survey (Table 1). About half of participants were male (51% phone, 49% online). The majority of participants smoked cigarettes every day (72%, 59%) and had ever used an e-cigarette or other vaping device (69%, 76%).

**Table 1:** Participant characteristics.

	Phone	Online
	*n* = 1,151	*n* = 1,561
	Weighted %	%
Age (years)		
18–25	14.2	19.2
26–34	20.4	41.9
35–44	17.8	20.9
45–54	25.0	10.9
55–64	14.9	6.4
65+	7.6	0.9
Mean (SD)	43 (15)	35 (13)
Male	50.7	49.2
Gay, lesbian, or bisexual	4.5	13.0
Race		
White	66.7	85.2
Black	21.9	7.2
Native American	2.9	1.1
Asian	0.9	3.5
Other	7.6	3.1
Hispanic	10.1	8.0
Lower numeracy	38.3	10.9
Education		
< High school	16.1	1.6
High school graduate or equivalent	38.4	16.5
Some college	25.9	34.5
Associate’s degree	10.2	13.2
College degree	7.2	27.8
Master’s degree	1.6	4.9
Professional or doctoral degree	0.5	1.5
Income, annual		
$0 - $24,999	41.0	25.1
$25,000 - $49,999	32.9	35.9
$50,000 - $74,999	13.4	23.1
$75,000 - $99,999	5.2	9.9
$100,000 or more	7.4	5.9
Poor mental health	21.3	10.2
Currently smoked “light” cigarettes	37.1	44.2
Smoking frequency		
Some days	28.2	40.9
Every day	71.8	59.1
Intent to quit within 6 months	48.2	45.2
E-cigarette use (ever use)	69.1	76.0

*Note*. Phone survey % weighted. Missing data ranged from 0% to 5%.

### Past brand switching

In the phone sample, 43% of respondents reported having switched cigarette brands or styles in the past to reduce health risk (Table 2). In adjusted analyses, older adults were more likely than younger adults to have switched brands (OR = 3.07, 95% CI = 1.50, 6.31) as were women compared to men (OR = 1.68, 95% CI = 1.09, 2.59) (Table 3). Past switching was also more likely among those who currently smoke “light” cigarettes (OR = 2.05, 95% CI = 1.30, 3.24).

**Table 2:** Unadjusted correlates of having switched cigarette brands to reduce health risk.

	Phone (*n* = 1,151)				Online (*n* = 1,561)			
	*n*/total	%	OR	(95% CI)	*n*/total	%	OR	(95% CI)
**Overall**	505/1151	43	-	-	444/1561	28	-	-
**Participant characteristics**								
Age (years)								
18–25 (Ref)	44 / 175	21	1.00	-	54 / 299	18	1.00	-
26+	461 / 976	46	3.18	(1.62, 6.26)*	390 / 1262	31	2.03	(1.48, 2.79)**
Sex								
Male (Ref)	242 / 597	37	1.00	-	185 / 768	24	1.00	-
Female	263 / 554	49	1.67	(1.08, 2.58)*	259 / 793	33	1.53	(1.22, 1.91)*
Sexual orientation								
Straight or heterosexual (Ref)	477 / 1073	43	1.00	-	380 / 1358	28	1.00	-
Gay, lesbian, or bisexual	22 / 65	31	0.58	(0.25, 1.32)	64 / 203	32	1.19	(0.86, 1.63)
Race								
White (Ref)	357 / 793	44	1.00	-	384 / 1330	29	1.00	-
Black or African American	98 / 235	39	0.82	(0.48, 1.41)	24 / 112	21	0.67	(0.42, 1.07)
Other	50 / 123	41	0.91	(0.47, 1.73)	36 / 119	30	1.07	(0.71, 1.61)
Hispanic								
No (Ref)	471 / 1075	42	1.00	-	417 / 1433	29	1.00	-
Yes	33 / 75	52	1.51	(0.75, 3.02)	27 / 125	22	0.67	(0.43, 1.04)
Lower numeracy								
No (Ref)	306 / 708	43	1.00	-	382 / 1390	27	1.00	-
Yes	199 / 441	42	0.96	(0.62, 1.49)	62 / 170	36	1.52	(1.09, 2.12)*
Less than high school education								
No (Ref)	235 / 544	40	1.00	-	371 / 1278	29	1.00	-
Yes	270 / 605	46	1.27	(0.82, 1.96)	73 / 283	26	0.85	(0.63, 1.14)
Low income (<$50,000/year)								
No (Ref)	109 / 283	42	1.00	-	171 / 607	28	1.00	-
Yes	396 / 868	43	1.03	(0.64, 1.68)	273 / 952	29	1.03	(0.82, 1.29)
Poor mental health								
No (Ref)	417 / 952	44	1.00	-	402 / 1399	29	1.00	-
Yes	88 / 198	38	0.79	(0.45, 1.39)	42 / 158	27	0.90	(0.62, 1.30)
**Tobacco product use**								
Smoked “light” cigarettes								
No (Ref)	296 / 753	35	1.00	-	183 / 871	21	1.00	-
Yes	209 / 395	56	2.32	(1.47, 3.66)*	261 / 689	38	2.29	(1.83, 2.87)**
Smoking frequency								
Some days (Ref)	129 / 322	38	1.00	-	149 / 638	23	1.00	-
Every day	376 / 829	45	1.33	(0.81, 2.19)	295 / 923	32	1.54	(1.23,1.94)*
Intent to quit in 6 months								
No (Ref)	245 / 609	40	1.00	-	210 / 855	25	1.00	-
Yes	256 / 528	46	1.27	(0.82, 1.97)	234 / 706	33	1.52	(1.22, 1.90)*
E-cigarette use (ever use)								
No (Ref)	145 / 360	38	1.00	-	78 / 374	21	1.00	-
Yes	360 / 791	45	1.36	(0.86, 2.15)	365 / 1184	31	1.69	(1.28, 2.23)*

*Note*. OR = odds ratio; CI = confidence interval; Ref = reference category. Phone survey %, OR, and CI weighted. Sample sizes for bivariate correlates varied; missing data ranged from 0% to 5%.

*p < .05

**p < .001

**Table 3:** Adjusted correlates of having switched cigarette brands to reduce health risk.

	Phone (*n* = 1,132)		Online (*n* = 1,556)	
	OR	(95% CI)	OR	(95% CI)
**Participant characteristics**				
Age (years)				
18–25 (Ref)	1.00	-	1.00	-
26+	3.07	(1.50, 6.31)*	1.73	(1.24, 2.42)*
Sex				
Male (Ref)	1.00	-	1.00	-
Female	1.68	(1.09, 2.59)*	1.37	(1.08, 1.72)*
Lower numeracy				
No (Ref)	1.00	-	1.00	-
Yes	0.93	(0.59, 1.48)	1.72	(1.21, 2.44)*
**Tobacco product use**				
Smoked “light” cigarettes				
No (Ref)	1.00	-	1.00	-
Yes	2.05	(1.30, 3.24)*	2.25	(1.78, 2.84)**
Every day smoker				
No (Ref)	1.00	-	1.00	-
Yes	1.18	(0.72, 1.92)	1.59	(1.24, 2.04)*
Intent to quit in 6 months				
No (Ref)	1.00	-	1.00	-
Yes	1.29	(0.82, 2.01)	1.52	(1.20, 1.92)*
E-cigarette use (ever use)				
No (Ref)	1.00	-	1.00	-
Yes	1.44	(0.90, 2.30)	1.62	(1.20, 2.16)*

*Note*. Adjusted model contained all correlates statistically significant (*p* < .05) in unadjusted models. OR = odds ratio; CI = confidence interval; Ref = reference category. Phone survey %, OR, and CI weighted. Analyses excluded 19 participants from the phone survey and 5 participants from the online survey with missing data on the correlates or outcome.

*p < .05

**p < .001

In the online sample, 28% of smokers reported having switched brands to reduce health risk (Table 2). As in the phone sample, in adjusted analyses, older adults (OR = 1.73, 95% CI = 1.24, 2.42), females (OR = 1.37, 95% CI = 1.08, 1.72), and smokers of “light” cigarettes (OR = 2.25, 95% CI = 1.78, 2.84) were more likely to report past brand switching (Table 3). In addition, smokers with lower numeracy, who had tried e-cigarettes, who smoked daily, and who wanted to quit smoking in the next 6 months were also more likely to have switched brands (all *p* < .05).

### Susceptibility to brand switching

The majority of smokers in the phone and online surveys were susceptible to brand switching if they were to learn that their cigarettes had a lot more of a particular chemical than other brands (median across experimental conditions 76%; range 61–92%; Table 4). In the phone experiment, information about the amount of lead, formaldehyde, arsenic, and ammonia led to higher susceptibility to brand switching than information about nicotine (all *p* < .05; Table 4); carbon monoxide and nicotine did not differ. In the online experiment, information about all five chemicals led to higher susceptibility to brand switching than nicotine (all *p* < .001).

**Table 4:** Effect of constituent level information on susceptibility to brand switching.

	Phone (*n* = 1,142)				Online (*n* = 1,558)			
	*n*/total	%	OR	(95% CI)	*n*/total	%	OR	(95% CI)
**Constituent**								
Nicotine (Ref)	115 / 188	61	1.00	-	187 / 265	71	1.00	-
Carbon Monoxide	135 / 193	70	1.48	(0.97, 2.26)	213 / 243	88	2.96	(1.86, 4.71)**
Lead	120 / 165	73	1.69	(1.08, 2.66)*	226 / 251	90	3.77	(2.31, 6.16)**
Formaldehyde	152 / 199	76	2.05	(1.32, 3.19)*	223 / 254	88	3.00	(1.90, 4.75)**
Arsenic	138 / 181	76	2.04	(1.30, 3.20)*	265 / 289	92	4.61	(2.81, 7.55)**
Ammonia	165 / 216	76	2.05	(1.34, 3.16)*	223 / 256	87	2.82	(1.80, 4.43)**

*Note*. OR = odds ratio; CI = confidence interval; Ref = reference category. Analyses excluded 9 participants from the phone survey and 3 participants from the online survey with missing data on the outcome.

*p < .05

**p < .001

## Discussion

Switching to a cigarette brand or style perceived as less harmful can give the false impression of having reduced the risk of harm and in this way encourage people to continue smoking. In two large US samples, many smokers had switched cigarette brands in the past to reduce their health risk. Many smokers also reported being susceptible to brand switching if they found out that their cigarette brand had a lot more of a particular chemical than other cigarettes. This finding was robust across two samples and across several chemicals (nicotine, carbon monoxide, lead, formaldehyde, arsenic, and ammonia). Information about lead, formaldehyde, arsenic, and ammonia led to more susceptibility to switch brands than nicotine, although susceptibility to brand switching was high (over 60%) for all chemicals.

We found that brand switching was more prevalent among older smokers, building on prior research demonstrating this association.[10, 22, 23] Older adults may be more concerned about health risks and may have had more time to switch. They also may be more likely to believe that “light” cigarettes are less harmful because of exposure to misleading advertising of low-yield cigarettes (now effectively banned in the US).[24–26] Therefore, older adults may have engaged in brand switching specifically to reduce their likelihood of smoking-related harms. We also found that women were more likely to have switched brands, perhaps because women are generally more attentive to and likely to take action regarding their health,[27, 28] and thus may have been more likely to switch brands to lower health risks. Furthermore, we found that those who currently smoke “light” cigarettes were more likely to have switched brands in the past. It is possible that these smokers had switched to “light” cigarettes in the past to lower their health risks, and they were maintaining this switch.[22]

The finding that the majority of smokers were susceptible to switching brands in response to chemical information is concerning. FDA is tasked with presenting information to the public about the quantities of chemicals in cigarette smoke by brand and sub-brand.[6] However, our study suggests that even qualitatively describing that one cigarette has more of a particular chemical may mislead smokers and steer them toward brand switching. Thus, there is a real risk of repeating the mass deception in decades past caused by the public disclosure of tar yields of cigarettes and the misleading marketing of cigarettes as “light.”[24–26] Unless a meaningful difference in harm is found among brands, governments and other producers of communication campaigns or regulatory disclosures should avoid presenting chemical information that allows for comparing brands. Instead, to avoid misleading the public, messages should emphasize that toxic amounts of these chemicals are in all cigarettes without listing specific quantities. Future studies should extend the current findings by examining the impact of chemical information on actual brand-switching behavior and on other potential unintended consequences, such as increased interest in “natural” and “organic” cigarettes.[29–33]

Our study’s strengths include the use of a nationally representative dataset in the phone survey, inclusion of large numbers of smokers, and replication of most findings across two samples. Limitations of the study include the use of cross-sectional data that largely prevents causal inferences about the association between correlates and past brand switching. The generalizability of the results to other populations (e.g., among youth) and settings (e.g., outside the US) remains unknown. In the correlational analyses, we observed more statistically significant findings in the online sample. These differences could be attributable to mode effects (e.g., the online survey allowed participants to respond to items at their own pace) or to differences in the educational and smoking characteristics of the samples. Our surveys assessed brand switching for health reasons but not for others such as to save money. Relatedly, our measures did not differentiate between switching to a new brand or within a brand, as we considered any switching that occurred in order to reduce health risks to be problematic. However, some research has shown that the two are distinct behaviors with different correlates, and these could be examined in future studies.[34]

Brand switching due to inaccurate perceptions of comparative risk may lead to less quitting and worse public health outcomes. Our study shows that many smokers have previously switched cigarette brands with the goal of reducing health risks. The majority of smokers would switch brands in the future if they learned their cigarettes had more of a harmful chemical than other brands. Communications about the harmful chemicals in cigarettes should work to reduce previous misperceptions and prevent new ones.

## Supporting information

S1 Appendix Survey items for study. (DOCX)
