# Supplementary material for: Brand switching and toxic chemicals in cigarette smoke: A national study
Source: PLoS One. 2018 Jan 11;13(1):e0189928. doi: 10.1371/journal.pone.0189928 (PMC5764241; doi:10.1371/journal.pone.0189928)
Supplement: S1 Appendix — (DOCX) [file pone.0189928.s001.docx]

**S1 Appendix.** **Survey items for study**

Survey items included in the paper are below. Phone and Online surveys used the same measures, with small adjustments as needed for the online survey. Additional survey details can be found elsewhere[1, 2].

| **Construct** | **Item** | **Response scale** | **Source** |
| --- | --- | --- | --- |
| Past brand switching | The next questions are about switching cigarette brands or cigarette styles, like from regulars to lights. Have you ever switched to another cigarette brand or style to reduce your health risk? | 0= No  1= Yes | New item |
| Susceptibility to brand switching | What if you learned that the cigarettes you smoke have a lot more [chemical] than other cigarettes? How likely would you be to switch to another cigarette brand or style?  Would you say…  [chemical= randomize to 1 of 6 chemicals: nicotine, carbon monoxide, lead, formaldehyde, arsenic, or ammonia] | 1 = You might switch,  2 = You’d definitely switch, or  3 = You wouldn’t switch? | New item |
| Cigarette use | Have you smoked at least 100 cigarettes in your entire life? | 0= No  1= Yes | Adapted from 2013 BRFSS[3] |
| Smoking frequency | Do you now smoke cigarettes every day, some days, or not at all? | 1= Every day  2= Some days  3= Not at all | Adapted from 2013 BRFSS[3] and PATH[4] |
| “Light” cigarettes | Do you usually smoke regular, light, or ultra light cigarettes? You might also know them by the pack color, like red, gold, or silver. | 1= Regular (or full flavor or red or black)  2= Light (or mild)  3= Ultra light  4= Gold  5= Silver  6= Blue  7= Other (specify)___________  8= I don’t have a usual style | New item |
| Intentions to quit smoking in 6 months | Are you planning to quit smoking… | 1= Within the next month,  2= Within the next 6 months,  3= Sometime in the future beyond 6 months,  4= Or are you not planning to quit? | Adapted from PATH[4] |
| Prompt | The next few questions are about electronic or e-cigarettes and other vaping devices, such as e-hookah and vape pens. Popular brands include Blu, Vuse, NJOY, and Flavor Vapes. |  | New item |
| E-cigarette use (ever use) | Have you ever used an e-cigarette or other vaping device, even one or two times? | 0= No  1= Yes | New item |
| Age | How old are you? | _______ years | Adapted from 2010 Census[5] |
| Sex | Are you… | 1= Male, or  2= Female? | Adapted from 2010 Census[5] |
| Sexual orientation | The next question is about your sexual orientation. Do you consider yourself to be… | 1= Straight or heterosexual  2= Gay or lesbian, or  3= Bisexual?  4= Other | Adapted from Williams Institute, 2009[6] |
| Race | Which one of these groups would you say best represents your race? | 1= White  2= Black or African American  3= American Indian or Alaska Native  4= Asian, or  5= Pacific Islander  6= Other | Adapted from 2031 BRFSS[3] |
| Ethnicity | Are you of Hispanic, Latino, or Spanish origin? | 0= No  1= Yes | Adapted from 2010 Census[5] |
| Numeracy | In general, which of these numbers shows the biggest risk of getting a disease? | 1= One in 100  2= One in 1000  3= One in 10 | Adapted from Lipkus et al., 2001[7] |
| Education | What is the highest degree or level of school you have completed? | 0= No schooling completed  1= Nursery school to 4^th^ grade  2= 5^TH^ or 6^TH^ grade  3= 7^TH^ or 8^TH^ grade  4= 9^TH^ grade  5= 10^TH^ grade  6= 11^TH^ grade  7= 12^TH^ grade - no diploma  8= High school graduate - high school diploma or the equivalent (for example: GED)  9= Some college credit, but less than 1 year  10= 1 or more years of college, no degree  11= Associate’s degree (for example: aa, as)  12= Bachelor’s degree (for example: ba, ab, bs)  13= Master’s degree (for example: MA, MS, MEng, MEd, MSW, MBA)  14= Professional degree (for example: MD, DDS, DVM, LLB, JD)  15= Doctorate degree (for example: PhD, EdD)] | Adapted from 2010 Census[5] |
| Income | Would you say that your total annual household income is… | 1= Below $25,000 per year  2= Between $25,000 and $49,999 per year  3= Between $50,000 and $74, 999 per year  4= Between $75,000 and $100,000 per year  5= Above $100,000 per year | Adapted from 2010 Census[5] and 2013 BRFSS[3] |
| Mental Health | In general, would you say your mental health is… | 1= Excellent  2= Very Good  3= Good  4= Fair, or  5= Poor? | Adapted from PATH[4] |

**S1 References**

1. Boynton MH, Agans RP, Bowling JM, Brewer NT, Sutfin EL, Goldstein AO, et al. Understanding how perceptions of tobacco constituents and the FDA relate to effective and credible tobacco risk messaging: A national phone survey of U.S. adults, 2014-2015. BMC Public Health. 2016;16:516. Epub 2016/06/24. doi: 10.1186/s12889-016-3151-5. PubMed PMID: 27333921; PubMed Central PMCID: PMCPMC4918079.

2. Brewer NT, Morgan JC, Baig SA, Mendel JR, Boynton MH, Pepper JK, et al. Public understanding of cigarette smoke constituents: three US surveys. Tob Control. 2016. Epub 2016/12/08. doi: 10.1136/tobaccocontrol-2015-052897. PubMed PMID: 27924009.

3. Centers for Disease Control. 2013 Behavioral risk factor surveillance system questionnaire. <http://www.cdc.gov/brfss/questionnaires/pdf-ques/2013%20brfss_english.pdf>. Accessed on 21 Jan 2016. 4. National Institutes of Health, Instruments for Wave 2 of the PATH Study. <http://www.reginfo.gov/public/do/PRAViewIC?ref_nbr=201407-0925-004&icID=212557>. Accessed on 21 Jan 2016. 5. Centers for Disease Control and Prevention. Current cigarette smoking among adults - United States, 2011. MMWR Morb Mortal Wkly Rep. 2012;61(44):889-94. 6. The Williams Institute. Best practices for asking questions about sexual orientation on surveys. <http://williamsinstitute.law.ucla.edu/wp-content/uploads/SMART-FINAL-Nov-2009.pdf>. Accessed on 21 Jan 2016. 7. Lipkus IM, Samsa G, Rimer BK. General performance on a numeracy scale among highly educated samples. Med Decis Making. 2001;21(1):37-44.
